# Schoolwide Positive Behavior Interventions and Support in Portuguese Schools: An Exploratory Study

**DOI:** 10.3390/bs16030344

**Published:** 2026-02-28

**Authors:** Marisa Carvalho, Rosário Serrão, Helena Azevedo, Joana Cruz, Lurdes Veríssimo

**Affiliations:** 1 Research Centre for Human Development, Faculty of Education and Psychology, Universidade Católica Portuguesa, 4169-005 Porto, Portugal; rscunha@ucp.pt (R.S.); lverissimo@ucp.pt (L.V.); 2 Department of Social and Behavioral Sciences, University of Maia, 4475-690 Maia, Portugal; hazevedo@umaia.pt; 3 Psychology for Development Research Center, Instituto de Psicologia e Ciências da Educação, Universidade Lusíada Porto, 4169-005 Porto, Portugal; joanacruz@por.ulusiada.pt

**Keywords:** school environments, positive behavior, school violence, prevention, Schoolwide PBIS, exploratory study

## Abstract

Schoolwide Positive Behavior Interventions and Support (Schoolwide PBIS) has been identified as a framework that supports schools in promoting positive behavior and preventing and managing behavior problems. The Decree-Law no. 54/2018 in Portugal leads schools to implement multitiered support systems for all students, integrating learning, social–emotional, and behavioral dimensions. Schoolwide PBIS aligns with the aims and principles of this legislation. However, to our knowledge, studies have yet to map Schoolwide PBIS practices in Portuguese schools. This study presents the results from a survey that aimed to explore the practice of Schoolwide PBIS features in Portuguese schools from the perspectives of 375 school psychologists and to analyze self-reported knowledge regarding the Schoolwide PBIS framework and its implementation in schools. Results suggest a limited Schoolwide PBIS implementation in Portuguese schools, with a meager percentage of school psychologists reporting a higher level of knowledge about the framework and the majority mentioning knowing little or nothing about Schoolwide PBIS. The results evidence how complex and challenging the adoption and implementation of Schoolwide PBIS can be, although there is strong evidence of its efficacy and relevance in inclusive contexts.

## 1. Introduction

Schoolwide Positive Behavior Interventions and Support (Schoolwide PBIS) have been identified as a framework that supports schools in promoting positive behavior and preventing behavior problems (Horner et al., 2009, 2017; Sugai & Horner, 2006). It is an approach developed in the United States, aiming to guide schools in creating safe, secure, and inclusive learning environments (Sugai & Horner, 2009). It is defined as an evidence-based preventive framework (Horner et al., 2009; Horner & Macaya, 2018), contributing to higher prosocial skills, better academic performance, and lower office discipline referrals (Bradshaw et al., 2012; Horner et al., 2009; Wienen et al., 2019), as well as having positive effects on students with high-risk trajectory in schools (Sørlie et al., 2018). Schoolwide PBIS has also been found to positively affect school climate dimensions (e.g., safety perception, teacher–student relationships) (Kubiszewski et al., 2023). There is sound evidence supporting Schoolwide PBIS implementation across the United States as well as in other countries such as Cyprus (Michael et al., 2024), Netherlands (Nelen et al., 2019), and Norway (Sørlie & Ogden, 2015). However, there is scarce work on introducing and implementing Schoolwide PBIS in other cultural contexts than the USA (Nelen et al., 2019). With the growing interest in multitiered approaches as effective ways to offer more inclusive educational contexts, specifically in Schoolwide PBIS as an effective framework to address behavior, the need for research about implementing it in other countries is increasing (Knoster, 2018).

The official introduction of multitiered approaches in Portugal occurred in 2018 with the publication of the inclusive education law (Decree-Law no. 54/2018). This law preconizes the importance of shifting from special education paradigm/remediate approaches to focusing the human resources of the school on preventive approaches for all students (Cruz et al., 2023), following a multitiered system of support (MTSS) framework (Alves et al., 2020) and motivating schools to implement this approach in all areas of development: learning, social and emotional, and behavioral. MTSS framework inspired the development of the legislation, which is used as a broad framework for learning and behavior support. Schoolwide PBIS, as a multitiered system for behavior support, is reflected in it through some shared key features (e.g., prevention-oriented; continuum intervention spanning universal, targeted, and intensive tiers; monitoring and data-based decision-making) and other specific features (e.g., emphasis on social behavior outcomes and universal interventions; assessment often indirect, using existing sources such as discipline data). Before the law, the implementation of Schoolwide PBIS in Portugal was scarce or almost inexistent, with some schools testing it to reduce indiscipline and other behavior problems (e.g., Carvalho et al., 2016, 2017). However, studies have yet to be developed in this country regarding Schoolwide PBIS implementation and effectiveness. In line with other studies (Michael et al., 2024; Nelen et al., 2019), this study aimed to gain more insights into the Schoolwide PBIS implementation processes in settings that do not necessarily fit in values rooted in the U.S. context. 

In Portugal, school psychologists are expected to intervene in three main domains, specifically psychological support and counseling; development of the educational community’s relational system; and vocational development (Direção-Geral da Educação & Ordem dos Psicólogos Portugueses, 2024). This study is particularly relevant, considering that, within the domain of psychological support and counseling, school psychologists are expected to implement interventions and provide support for positive discipline practices. Furthermore, recent research (Gaspar et al., 2022) indicates that mental health and behavioral problems among students in Portugal are increasing, which can negatively affect cognitive development, academic performance, and social relationships. Schools represent a key setting for early identification and prevention of mental health problems, as well as for promoting students’ socio-emotional skills and wellbeing. In this context, Portuguese school psychologists have been called upon to prioritize preventive and promotional practices, systems-level practices grounded in a MTSS framework, shifting beyond predominantly individual assessment and counseling toward interventions involving contexts and interactions that enhance the inclusion and student wellbeing (Carvalho et al., 2024; Direção-Geral da Educação & Ordem dos Psicólogos Portugueses, 2024).

Despite the well-established value of schoolwide preventive approaches, implementing frameworks such as MTSS and Schoolwide PBIS requires acknowledging schools as multidimensional, complex, and adaptive institutions in which change emerges through interactions among interdependent subsystems rather than linear plans (Kershner & McQuillan, 2016). Accordingly, implementation consistency depends not only on policy mandates or technical design, but also on alignment between visible organizational structures and routines (e.g., roles, decision rules, time allocation, data routines) and less visible cultural and relational processes that shape shared meaning, and collective responsibility for enactment across settings and staff (Michael et al., 2024; Nelen et al., 2019). Sustaining improvement, in turn, requires change that becomes embedded in everyday practice and organizational routines, maintained over time, and progressively owned by local actors (Hubers, 2020). In this context, collaborative professionalism supports the sustainability of the educational innovations, by focusing on collaboration grounded in shared responsibility and collective efficacy (Hargreaves & O’Connor, 2018). In Portugal, this systems-oriented stance aligns with national guidance that positions school psychologists as key agents of preventive, whole-school change within multitiered support, working with leadership, teachers, families, and multidisciplinary teams to reduce barriers to learning and inclusion and promote positive behavior and school climate (Carvalho et al., 2024; Direção-Geral da Educação & Ordem dos Psicólogos Portugueses, 2024).

### 1.1. Schoolwide Positive Behavior Interventions and Support

Faced with the increase in behavior problems, schools tend to increase monitoring and supervision, redefine and reinforce rules and sanctions, expand the continuum of punitive consequences, and reinforce consistency in the reactions of different actors in situations of disruption (Sprick et al., 2002; Sugai & Horner, 2002). Schools tend to act reactively, with low tolerance mechanisms towards manifestations of indiscipline, based on the idea that increasing punishments for repeated indiscipline behavior will “teach” the student that their behavior is unacceptable (Sugai & Horner, 2006). However, research evidence shows that reactive responses contribute only to the immediate and short-term reduction in behavior problems. Its isolated application is ineffective in consolidating a positive school climate that prevents the development and occurrence of disruptive behaviors. In the long term, reactive and remedial actions promote a false sense of security, inadvertently reinforce antisocial behaviors such as aggression and vandalism, contribute to increased dropout rates, and reduce learning opportunities (Öğülmüş & Vuran, 2016; Skiba & Peterson, 2000; Sprick et al., 2002; Sugai & Horner, 2002, 2006). The school must, therefore, prioritize preventive strategies such as providing direct and contextualized instruction of prosocial skills, increasing opportunities for learning positive behaviors and success, and promoting a positive school climate (Sugai & Horner, 2002, 2006). The literature advocates that schools use proactive models of behavior management, which focus on evidence-based prevention strategies (e.g., Algozzine et al., 2011; Kutash et al., 2007; Sprague & Horner, 2006; Sugai & Horner, 2002, 2006).

Schoolwide PBIS provides a framework for schools to apply evidence-based behavioral strategies across three tiers of behavior support and interventions (Horner et al., 2009, 2017). Schoolwide PBIS main components are (i) high quality and scientifically validated instruction/teaching (i.e., positive behaviors are instructed directly and in the context); (ii) preventive focus on behavioral problems as a more effective and efficient response than remediation; (iii) MTSS (i.e., all students receive universal support; some students receive more structured and intensive teaching and feedback; and a few students will need highly individualized and focused support); (iv) using universal screenings to inform decision-making; and (v) continuous monitoring of progress. All of these components occur in a whole school approach paradigm, meaning that all school community is involved, and prosocial skills are taught not only in the classroom setting but in all places of the school (Horner & Macaya, 2018). Schoolwide PBIS implementation engages each school in a collaborative approach, considering the specific needs of each context and structuring the behavior support into three tiers. The first fully focused on prevention and involves “defining, teaching, monitoring, and rewarding a small set of behavioral expectations for all students across non-classroom and classroom settings” (Horner et al., 2009, p. 134). Alongside this, students considered to need more instruction than the one being provided in tier one are engaged in tier two support, for instance, with more hours for explicit instruction of skills and opportunities to practice the skills, and often including communication with families to increase students’ success (Harlacher & Rodriguez, 2017). This support can be provided in a group format or individually. However, for a small percentage of students, it is expected that more than tiers one and two interventions and support are needed to meet their behavioral needs. So, these students are engaged in tier three with individualized support, resourcing, for instance, to Functional Behavioral Assessment and multidisciplinary interventions (Harlacher & Rodriguez, 2017). The instruction at this level is more intense and frequent than in tier two (Sugai & Horner, 2002).

Schoolwide PBIS implementation is challenging as it is not a curriculum or a manual approach that can be used step by step (Horner & Macaya, 2018), and it takes time, around three years, for full implementation and system change (Horner et al., 2009). Additionally, successful and sustainable transfer and implementation of Schoolwide PBIS requires addressing cultural fit and implementation with fidelity. Cultural or contextual fit is “the match between strategies, procedures, or elements of an intervention and the values, needs, skills, and resources available in a setting” (Horner et al., 2014, p. 1). Therefore, cultural characteristics of a country, educational organization (e.g., law, professionals, training), and specific aspects of the school (e.g., size, resources, community) must be accounted for Schoolwide PBIS implementation. Additionally, the perspectives of those involved in implementing the intervention (e.g., teachers, psychologists) and those receiving it (e.g., students and families) should be considered (Horner et al., 2010; Nelen et al., 2019; Sugai et al., 2011). Adaptations in the Schoolwide PBIS procedures are required to fit the specific context and ensure successful implementation. However, fidelity is also a condition for Schoolwide PBIS implementation. Fidelity refers to the adherence to the original theoretical model, research protocol, and practical guidelines developed and agreed upon during the design process of Schoolwide PBIS implementation (Michael et al., 2024). The Tiered Fidelity Inventory (TFI; Algozzine et al., 2019) is an instrument used to assess the extent to which Schoolwide PBIS is implemented with fidelity. It is considered a valid, reliable, and efficient measure to analyze the extent to which Schoolwide PBIS core features are applied at the school (McIntosh et al., 2016). Some of these are: (i) having an established multidisciplinary team within the school; (ii) having behavioral expectations by setting the school clearly defined; (iii) having intentional moments for teaching these behavior expectations (in the classroom and across the school settings); (iv) having problem behaviors identified and defined, and a clear policy for how to address those behaviors; (v) having a professional development plan for supporting staff in the incorporation of Schoolwide PBIS features; (vi) having the involvement of the school community; (vii) collecting discipline data and making decisions based on that data.

### 1.2. The Present Study

Alongside other studies (Michael et al., 2024; Nelen et al., 2019), this study addresses the international need to expand positive behavior support in educational systems worldwide. Portugal is usually recognized for its inclusive education law and its educational system improvements in the last decade (OECD, 2022). Multitiered approaches are one of the most referred changes brought by the inclusive education paradigm dominant in the Portuguese educational system (Alves et al., 2020; Cruz et al., 2023). The recommended use of multitiered approaches, specifically Schoolwide PBIS, by Portuguese law represents an opportunity for more schools to implement Schoolwide PBIS. However, diverse challenges exist, such as the limited preparation of schools and professionals to the requirements of the inclusive education law (Alves et al., 2020), unfamiliarity with MTSS approaches such as Schoolwide PBIS (Cruz et al., 2023), inexperienced professionals in implementing this type of approaches (Carvalho et al., 2024), and prevalence of punitive and reactive strategies to address indiscipline and problem behaviors in Portugal (Carvalho et al., 2017). In the Portuguese educational context, school discipline is supported by a strongly formalized regulatory framework that prioritizes the reporting of infractions and the application of corrective and punitive measures. For instance, the Portuguese Student statute (Law 51/2012) states several procedures for reporting disciplinary occurrences and sets a continuum of corrective and sanctioning practices, which may contribute to a school culture focused on correction behavioral violations rather than preventing them and reinforcing expected behavior. In this context, the absence of studies that map and describe Schoolwide PBIS practices in Portuguese schools’ blind aspects regarding the successful transfer and implementation of this approach in this specific context.

The primary purpose of this study was to explore the implementation of Schoolwide PBIS features and characteristics in Portuguese schools through the lens of school psychologists. The school psychologists in Portugal are the professionals within school contexts with the capacity to provide applied behavioral expertise and professional development interventions (Breia et al., 2024; Ordem dos Psicólogos Portugueses, 2017), being an essential element for Schoolwide PBIS implementation (Algozzine et al., 2019). They are mandatory members of multidisciplinary teams focused on inclusive education (i.e., Decree-Law no. 54/2018 postulates that each school must have an inclusive education multidisciplinary team, being the school psychologist one of the permanent members of this team) and often assume a coordination role in those teams. As many school psychologists have not been trained in Schoolwide PBIS, the Portuguese Ministry of Education has supported the professional development of school psychologists with specific training on MTSS, including schoolwide positive behavior interventions and supports (Carvalho et al., 2024). Therefore, school psychologists are pivotal professionals in schools regarding Schoolwide PBIS implementation. Their perspectives on this topic are of particular relevance to the objectives of this study.

The objectives were twofold:

(i) Explore the school practices that exist to promote positive behaviors from the perspectives of school psychologists;
(ii) Analyze the self-reported knowledge of school psychologists regarding the Schoolwide PBIS framework and its implementation in the school.

## 2. Method

### 2.1. Participants

Data were collected through 375 school psychologists from 375 schools in Portugal. This number of participating schools means that this study had a 75% response rate—75% of all public schools in Portugal mainland are represented. Each school psychologist was unique to one school. Most participants were female (*n* = 344, 91.7%), with a Master’s degree (*n* = 202, 53.9%) and up to five years of experience at their school (*n* = 151, 40.3%). The mean age of the participants was 45.30 years (SD = 8.99; see Table 1 for the participants’ sociodemographic data).

Participating schools were diverse and were in different geographic contexts. Most schools were located in semi-urban contexts (*n* = 152, 40.5%) and clustered (*n* = 317, 84.5%), indicating that they integrated several other schools with varying levels of education, ranging from early childhood to secondary. Most were regular schools (*n* = 245, 65.3%), but specific schools participated in the study, such as those integrated in disadvantaged social contexts, professional schools, and artistic schools. Most schools had between 1001 and 1500 students (*n* = 94, 25.1%; see Table 2).

### 2.2. Measures

*Sociodemographic and school-level characteristics questionnaire*

Sociodemographic characteristics included school psychologists’ age, gender, qualifications, domain of specialization in Psychology, and years of experience in the psychologist’s school. School-level characteristics regarding school context, level of teaching, type of school, and number of students were obtained.

*Mapping Positive behavior practices in Portuguese schools’ survey*

Survey development occurred in two stages. Stage 1 involved an extensive review of schoolwide positive behavior interventions and specific measures to access practices and scientific knowledge to implement this framework. The main resources were used as inspiration to develop the survey: The School-Wide Evaluation Tool (SET) (Horner et al., 2004) and the Schoolwide PBIS Tiered Fidelity Inventory (Algozzine et al., 2019), particularly the dimensions related to Tier One features. These resources were used to take ideas on item phrasing, but mostly to ensure the inclusion of all relevant features of Schoolwide PBIS, as well as support in organizing the different items. Stage 2, hence, consisted of selecting items and adapting them to the Portuguese context (e.g., district support was not considered as this is not applicable to our context). A self-report survey was then created to assess school psychologists’ self-perception regarding practices of Schoolwide PBIS in Portuguese schools, as well as perceived knowledge about the conceptual framework. The survey was organized stating and focusing on the most critical Schoolwide PBIS features and deconstructing the framework into concrete actions, as we expected that schools might only be applying parts of the Schoolwide PBIS components and not the entire framework. Stage 3 involved asking school psychologists with knowledge on Schoolwide PBIS and researchers in the area with knowledge on Portuguese educational context (including authors from this article that were not part of the initial development of the questionnaire) to provide feedback on the relevance of each item and its adequacy to the Portuguese context. Items were then slightly modified based on their feedback, which involved for instance adding examples in some items to support the understanding of participants about what was being asked. This stage helped ensure that these items were understood and accurately represented the leading practices, behaviors, and knowledge associated with implementing a Schoolwide PBIS framework in the school context.

Unlike the Schoolwide Evaluation Tool and the Schoolwide PBIS Tiered Fidelity Inventory, it was decided that a 4-point Likert scale response format focused on the perceived degree of implementation of each specific practice (each item) would be more useful in mapping the implementation of Schoolwide PBIS in Portuguese schools. Therefore, the following options were applied to access self-perception practices of Schoolwide PBIS in Portuguese schools: 1—Non-existent; 2—Emergent; 3—In progress; 4—Sustained. The option Do not know was also possible.

Ultimately, the survey consisted of two parts. The first part was made out of 28 items about eleven topics relevant to Schoolwide PBIS implementation without explicitly mentioning that those practices are part of Schoolwide PBIS, simply aiming to explore the level of implementation of each practice isolated. These items followed the organization proposed by Horner and colleagues (2004): (1) expectation defined (e.g., “Defines and disseminates expectations and examples of expected behaviors for school professionals in different settings of the school”; (2) behavioral expectations taught (e.g., “The expected social behaviors are taught directly and explicitly in the classroom”); (3) system for rewarding behavioral expectations (e.g., “Positive behaviors are recognized and valued”); (4) system for responding behavioral violations (e.g., “The school has clear procedures for responding to behavioral problems with students”); (5) shared responsibilities (e.g., “The school involves families in developing a vision oriented to the promotion of positive behavior”); (6) management (e.g., “The school has a team responsible for designing, implementing and monitoring positive behavior interventions”); (7) monitoring and evaluation (e.g., “The school collects and analyses periodically behavioral indicators”); (8) data-driven decision-making (e.g., “The school makes pedagogical decisions considering the analysis of the behavioral indicators”); (9) partnerships (e.g., “Partnerships are developed to support the design, implementation, and monitoring positive behavioral interventions”); (10) support to implementation (e.g., “Auxiliary staff receive training and/or ongoing support for the implementation of behavioral interventions and supports”); (11) projects, interventions, and support (e.g., “The school has specific interventions to respond to behavioral problems”).

The second part aimed to assess perceived knowledge about the conceptual Schoolwide PBIS framework. For this, a 5-point Likert scale response format was used (1—none; 2—little; 3—some; 4—good; 5—very good) for two items: “How familiar do you think you are with the Positive Behavioral Interventions & Supports (PBIS)/Schoolwide Positive Behavioral Interventions and Supports (Schoolwide PBIS) model?” and “How familiar do you think you are with the main features of the PBIS/Schoolwide PBIS model?”. In this case, we did not aim to assess school psychologists’ knowledge of each specific feature, but rather only their self-perception, considering the model as a whole.

### 2.3. Procedure

This study was assessed with positive approval by the Ethics Committee Technology, Social Sciences and Humanities of Universidade Católica Portuguesa (protocol code of the approval: CETCH2023-44; data of approval: 7 March 2023). Furthermore, a request was made to the Portuguese Ministry of Education to collect the data, and permission was provided.

The survey was disseminated through e-mail to all Portuguese school principals using a database with contact details of all public schools in mainland Portugal, in 500 schools. The e-mail was sent with the invitation to participate in the study and the request for referral to the school psychologist to complete the survey. There was no mandate to participate in the study; participation was fully voluntary. The school psychologist in Portugal is the leading professional within schools with the capacity to provide applied behavioral expertise and professional development interventions in this area necessary for Schoolwide PBIS implementation (Algozzine et al., 2019), being usually part of teams focused on inclusive education, and therefore, this was the critical professional within the school chosen to provide information for this research related to the level of implementation of Schoolwide PBIS.

All relevant information for the participants’ informed consent was presented before the beginning of the questionnaire. No information was requested that would allow us to identify each participant individually, ensuring the anonymity of all data. Participants were required to complete an anonymous self-report online survey, which included an instruction sheet and a consent form. They were assured of confidentiality and informed that their participation was voluntary. Scale administration occurred between September and December 2023.

### 2.4. Data Analysis

Due to the study’s exploratory nature, data were collected through Qualtrics and then downloaded into IBM SPSS 28.0 for descriptive analyses. These analyses enabled us to determine the level of Schoolwide PBIS implementation reported by schools’ psychologists and to identify the areas of strength and weakness (Debnam et al., 2012).

## 3. Results

### 3.1. School Practices to Promote Positive Behaviors

Results show that 84 (22.4%) schools have implemented projects and interventions inspired by the Schoolwide PBIS framework. The level of implementation differs among schools: 24 (27.9%) are at the emergent level, 47 (54.7%) are in progress, and 10 (11.6%) are at the sustained level.

Despite the low percentage of schools implementing the Schoolwide PBIS model, schools have adopted practices to promote positive behaviors. Table 3 shows the level of implementation of school practices to promote positive behaviors, represented by the number and percentage of schools in each category. Overall, results show a high implementation of practices to promote positive behaviors. However, a low percentage of schools have a team responsible for designing, implementing, and monitoring positive behavior interventions (24.8%) and a written document to guide these practices (22.4%) at a sustained level.

Most schools (over 50%) have practices in progress or sustained regarding defining and teaching behavioral expectations. Note that a considerable percentage of schools (19.5%) have not defined behavioral expectations for school professionals to establish and maintain a predictable, positive, and consistent behavior by all the adults, meaning that the professionals themselves may be unaware of transversal behavioral expectations in the school. This is even more important in a school, as many schools in Portugal, with high percentage of professionals turnover. Likewise, the percentage of schools that have not defined expected behavior for students is low (12.5%). Shared behavioral expectations by all professionals and students is a sine qua non element to promote an adequate school culture regarding behavior and interactions amongst the school community.

Most schools have sustained practices in responding to behavioral violations (55.2%) and consistently implement them (45.3%). Nevertheless, a lower percentage of schools incorporate a sustained system for rewarding behavioral expectations (25.9–37.3%).

Most schools involve all professionals, teachers, non-teachers, staff, families, and students in their practices to promote positive behavior. In-progress and sustained training and ongoing support are in place in most schools to support the implementation of positive behavior interventions. However, administrative staff are less involved in this training than teachers and auxiliary staff.

Considering monitoring and evaluation practices, results show that most schools collect and analyze behavioral indicators and disseminate these data among teachers, staff, students, and families. These data are used to drive the decision-making process related to organizational and pedagogical issues.

The results also indicate that many schools have sustained projects, interventions, and support to promote positive behaviors and manage and respond to behavior problems.

### 3.2. Knowledge of School Psychologists Regarding the Schoolwide PBIS Framework

Regarding self-reported knowledge of school psychologists, most recognized they have little or no knowledge of the Schoolwide PBIS model (57.3%) and its main features (60.5%). A low percentage of school psychologists reported a higher level of knowledge about the framework (7.4%) and its main characteristics (7.5%; see Table 4).

## 4. Discussion

Implementation of Schoolwide PBIS in non-U.S. educational settings is relatively unexplored (Knoster, 2018; Nelen et al., 2019). A successful implementation of Schoolwide PBIS depends on the cultural adaptation of its core features, notwithstanding reporting implementation fidelity (Michael et al., 2024). If the core features of Schoolwide PBIS should be constant across settings, its procedures are expected to vary according to context, putting those core features in place (Horner et al., 2014). Therefore, the present exploratory study aimed to describe how Schoolwide PBIS is being implemented in Portuguese schools. Specifically, we aimed to map the implementation of Schoolwide PBIS features in Portuguese schools from the perspectives of school psychologists and to analyze these professionals self-reported knowledge regarding the Schoolwide PBIS framework and its current implementation in schools.

Results show only 22.4% of schools implement projects and interventions inspired by the Schoolwide PBIS framework, and not all with a sustained implementation level. Surprisingly, Portuguese schools are referred to as having a limited Schoolwide PBIS implementation, considering that it is a recommended framework by Portuguese educational law (Decree-law no. 54/2018). Additionally, in recent years, particularly after the COVID-19 pandemic, school priorities included promoting mental health and well-being, social and emotional learning, and prosocial skills (Matos et al., 2023). In the specific case of Portugal, the Ministry of Education provided financial support to reinforce actions related to these priorities, such as training for school psychologists in the implementation of MTSS applied to learning, social and emotional development, and behavior (Carvalho et al., 2024) and through plans aimed to enhancing learning and social and emotional competences (Learning Recovery Plan 21|23 School+/Plano de Recuperação das Aprendizagens 21|23 Escola+). Therefore, considering these top-down efforts, it would be expected that more Portuguese schools would engage in Schoolwide PBIS implementation. Some explanations can be discussed.

Firstly, academic, behavioral, and socio-emotional needs are often seen as separate arenas and as such schools tend to implement shredded interventions for each specific area of development. Schools and their professionals focus on continuously changing and adapting to the student’s needs and, in the recent years, after the COVID-19 pandemic, Portuguese schools have focused on reducing psychopathological symptoms and promoting socio-emotional skills, rather than promoting positive behavior. A post-pandemic study in Portugal, with 8067 children and adolescents attending from preschool to 12th grade (“School Observatory: Monitoring and Action; Psychological Health and Well-being Study”, Matos et al., 2023) showed that a third of Portuguese students evidenced psychological distress and socio-emotional difficulties, a problem that worsens as schooling progresses, which justified the focus on mental health. Portuguese schools have also invested, in recent years, in promoting academic learning, incorporating RTI models, but unfortunately, not yet in an articulated way with Schoolwide PBIS models. For instance, the National Programme for Promoting School Success (PNPSE Report, Verdasca et al., 2022), implemented in Portugal, includes eight priority areas, namely Socio-emotional Intelligence and Personal Development, Family Involvement, Community Involvement, Reading and Writing Literacy, Multiculturalism and Citizenship, Tutoring and Mentoring, Digital Literacy, and Arts, Expressions, and Culture. No domain directly presupposes promoting positive behavior, and the main measures adopted focused on socio-emotional development and literacy promotion (PNPSE Report, Verdasca et al., 2022). Hence, although the literature highly recommends an integrated implementation of school efforts, considering all arenas of development—academic, social, emotional and behavioral (i.e., combining Schoolwide PBIS and RTI) (Santiago et al., 2024)—it seems that Portuguese schools are not yet approaching the implementation of MTSS efforts in this integrated way, giving privilege to academic learning and socio-emotional development due to recent needs within the school contexts, and possibly leaving aside an intentional preventive approach to the development of positive behavior. This is also aligned with the literature, as it is recognized that an integrated approach, though beneficial, is a highly complex task, which requires significant coordination efforts within schools that might be lacking in the Portuguese context (Santiago et al., 2024).

Secondly, the results indicate a higher implementation of procedures responding to behavioral violations than a rewarding and recognition of positive behaviors. This pattern is consistent with the predominant vision in Portuguese schools related to problem behaviors and how to manage these, anchored, over the years, in a punitive and reactive paradigm. This makes it more challenging to align school communities with Schoolwide PBIS core features and even engage different school community members in the relevance of promoting positive behavior rather than managing problematic behavior. This, allied with the need to support students’ learning and mental health after the COVID-19 pandemic, might help explain a low percentage of schools actively involved in Schoolwide PBIS implementation. Moreover, these results highlight and reinforce the need to strengthen Schoolwide PBIS preventive components through training and implementation support.

Thirdly, although MTSS framework is prescribed by the Ministry of Education, the reality is that there is a limited offer of professional training on multitiered approaches and even less specifically on Schoolwide PBIS. As previously mentioned, the Ministry of Education organized specific training for school psychologists, which included some time for group supervision, expecting these professionals to function as leaders and levers in starting Schoolwide PBIS implementation efforts locally. However, school psychologists are confronted with a high workload and barriers which prevent them from taking on leadership roles in the schools (Carvalho et al., 2024). Hence, other professionals also need to be trained and involved in the efforts to implement Schoolwide PBIS, such as administrators and teachers. In the USA, where Schoolwide PBIS is more widespread, there is a high involvement and support from districts, for instance, in the development of blueprints that support the dissemination of knowledge on the implementation of Schoolwide PBIS, but also continuous professional support. A recent study in the state of Oregon found that, particularly to combine Schoolwide PBIS with academic support, “a rigorous system of professional development and coaching appears necessary” (Chaparro et al., 2019). Another study also found that district support was seen as essential to maintain Schoolwide PBIS practices through the years in the schools, highlighting the importance of, for instance, coaches and district direct support to schools (George et al., 2018). Likewise in Portugal, professional development and support must be planned and thought out, including which professional development methods are most adequate, as strategies such as coaching seem to be relevant for a sustained implementation of MTSS efforts.

Likewise, the need for professional development in the Schoolwide PBIS approach also remains for school psychologists themselves, considering that 60.5% of the participants in this study—the majority—mentioned knowing little or nothing about this framework. Only a meager percentage of school psychologists reported a higher level of knowledge about the framework (7.4%) and its main characteristics (7.5%). Moreover, it is essential to note that a percentage of participants lack the knowledge about their school incorporating Schoolwide PBIS features, namely in monitoring and evaluation (e.g., if data are collected related to behavior—6.1%), data-driven decision making (6.7%), and partnerships with the community (8.0%). On the one hand, the literature posits school psychologists as essential professionals in supporting schools’ inclusive education practices based on whole-school approaches, such as the Schoolwide PBIS framework (Bartolo, 2015; Farrell, 2010; Jimerson et al., 2021), which is aligned with and reinforced in Portugal by the Decree-Law no. 54/2018 and the recommendations for the practice of school psychologists by the Ministry of Education (Breia et al., 2024) and the Portuguese Psychology Association (Ordem dos Psicólogos Portugueses, 2017). However, the present study’s results lead to questioning both what role do school psychologists have in their school related to behavior development and the effectiveness of the professional development efforts that have existed for school psychologists (Bloomfield et al., 2024; Carvalho et al., 2024). Indeed, the professional development opportunities might not have been sufficient to explicitly develop knowledge and skills in Schoolwide PBIS and did not cover all school psychologists in Portuguese schools. Furthermore, these findings reflect the relevance of a whole-school approach applied to the professional development of all educational agents as necessary to implement the Schoolwide PBIS framework in schools (Alves et al., 2020; Bloomfield et al., 2024). Implementing Schoolwide PBIS requires school psychologists to have leadership skills to engage teachers, non-teacher staff, families, and principals in school practices to promote positive behaviors and share a vision and responsibilities (Schaffer, 2021).

Several “pieces” of Schoolwide PBIS seem to be put in place in Portuguese schools, as school psychologists reported positively on implementation questions that we asked—several practices considered as “in progress” or “sustained”. This is, on its own, a good and encouraging result. Yet, simultaneously, school psychologists also reported on having rather limited knowledge of Schoolwide PBIS. Schoolwide PBIS shares its core features with other theories and frameworks regarding behavior development, particularly psychological approaches to manage problem behavior and prevent it. This might contribute to explaining why some practices are perceived to be put in place, even if they are unaware of their relation to Schoolwide PBIS. However, the Schoolwide PBIS framework highlights that it is the conjugation of all its core features that will make implementation effective over time. Consequently, there seems to be a need to increase knowledge on all core features of Schoolwide PBIS, and support schools in putting them into practice consistently, instead of only engaging in some practices. This can also contribute to increasing the buy-in of school community members in engaging in a purposeful whole-school strategy to promoting positive behavior.

One core aspect of the framework is the existence of a team, composed of members with leadership roles in the school, knowledge about school and community, and knowledge and skills about behavior support practices, to support the planning, implementation, and monitoring of Schoolwide PBIS (e.g., Algozzine et al., 2019). In the present study, only 24.8% of the schools reported having a team responsible for designing, implementing, and monitoring positive behavior interventions. However, in the specific case of Portugal, it is mandatory by law that all schools have a multidisciplinary team responsible for inclusive education (and in this scope, behavior issues), which is composed of diverse stakeholders (e.g., principal, teachers with leadership roles, special education teachers, psychologist). The political guidelines for these teams are recent (Pereira et al., 2018), and schools may need more time and support to create resources and knowledge to implement evidence-based practices to promote positive behavior interventions. As previously mentioned, often the focus of the multidisciplinary teams is on specific areas of development, mainly academic learning and implementing the necessary tiered support for all students. Again, we believe this study’s results point to the need to support schools in adopting a more integrated and less compartmentalized approach when implementing MTSS efforts. A clear cultural adaptation of the Schoolwide PBIS to the Portuguese school contexts would be that the already existing multidisciplinary team for inclusive education takes on the role of Schoolwide PBIS team simultaneously, instead of forcing schools to create a new team exclusively dedicated to the promotion of positive behavior.

Another critical feature of Schoolwide PBIS is the need to ensure continuous support for the professional development of staff. In the present study, 20.8% of teachers, 16.3% of auxiliary staff, and 14.9% of administrative staff reported receiving training and/or ongoing support for implementing behavior interventions and support. Hence, these professionals need more support and training to create a shared framework between the school staff. This enables the beginning of a whole-school focus on implementing Schoolwide PBIS (Alves et al., 2020; Cruz et al., 2023). Furthermore, only 23% of the Portuguese schools participating in this survey have a written document guiding school professionals’ practices for promoting positive behavior. The lack of clear guidelines within the school community may raise differences and problems in managing, monitoring, and evaluating Schoolwide PBIS practices. Only 32% of Portuguese schools sustainably define and disseminate expectations and examples of expected behaviors for students in different school settings. As recognized in previous studies (e.g., Royer et al., 2022), by defining expectations in collaborative processes, these expectations will be more likely to be taught to students and recognized, valued, and reinforced by all adults. Many schools that adopt these strategies do not have a structured Schoolwide PBIS framework but implement scarce and punctual strategies disconnected from the conceptual model.

The adoption and implementation of Schoolwide PBIS is complex and challenging as it is a whole-school approach that needs to address the cultural context and the organizational conditions (Horner & Macaya, 2018). Adopting Schoolwide PBIS requires one to three years of the whole school’s involvement, with organizational and leadership support and a well-trained team skilled in the Schoolwide PBIS framework, implementation science, and monitoring processes (Horner & Macaya, 2018). In Portugal, a remedial and reactive approach to problem behavior prevails, with crystallized systems for recording and managing indiscipline phenomena with a punitive focus and negligible permeability to a genuinely preventive approach and explicit teaching of expected behavior. In addition, school psychologists also tend to assume a clinical approach to behavior intervention instead of valuing a preventive framework (Mendes et al., 2015). Hence, it seems that one of the first steps to be taken in order to increase the implementation of Schoolwide PBIS is to increase the buy-in of all stakeholders by increasing their knowledge on the relevance of promoting positive behavior, its preventative benefits and its associations with all other arenas of development, engaging the school professionals in a more integrative approach when conducting MTSS efforts.

As previously mentioned, since the COVID-19 pandemic, there has been a shift in schools focusing on their role as providers of mental health support. One important argument to disseminate in schools is to consider the literature showing positive mental health outcomes on students in schools implementing Schoolwide PBIS. Schoolwide PBIS has been shown to effectively address externalizing problems in students; McIntosh et al. (2013) also found benefits for internalizing problems (which are the most common after COVID-19) by “adding evidence-based interventions for supporting internalizing needs within Schoolwide PBIS systems, providing professional development in identifying internalizing problems, and incorporating screening for internalizing problems into existing screening systems”.

Support for internalizing challenges within a Schoolwide PBIS framework can be enhanced by incorporating evidence-based interventions that address internalizing needs, providing professional development to identify internalizing problems, and integrating screening for internalizing problems into existing screening systems.

## 5. Conclusions

The present study highlights the novelty of Schoolwide PBIS in Portuguese schools. Although conceptual and educational policies directly suggest the need to implement this framework in schools, the results evidence the need for more schools to implement Schoolwide PBIS and the limited knowledge school psychologists report concerning this approach. Several practice recommendations can be suggested. Firstly, there is a need for increased professional development and training for school psychologists on the Schoolwide PBIS framework. Given the low percentage of psychologists with a high level of knowledge about Schoolwide PBIS, targeted training programs should be developed to enhance their understanding and skills in implementing this framework effectively (Bloomfield et al., 2024; Carvalho et al., 2024). Secondly, schools should establish and support multidisciplinary teams responsible for behavior interventions. Strengthening these teams can ensure better planning, implementation, and monitoring of Schoolwide PBIS initiatives (Algozzine et al., 2019).

Additionally, ongoing support and professional development should be provided for all school staff, including teachers, auxiliary staff, and administrative personnel. This support is essential for creating a shared understanding and cohesive implementation of Schoolwide PBIS across the whole school (Algozzine et al., 2019). Lastly, implementing Schoolwide PBIS requires a concerted effort from policymakers, school administrators, and educational stakeholders to create an environment that supports the comprehensive adoption of the framework (Horner & Macaya, 2018). In contrast, the academy may be essential in supporting and supervising schools’ efforts with evidence-based strategies.

Although this study provides an exploratory description of the implementation of Schoolwide PBIS features in Portuguese schools from the perspectives of school psychologists, some limitations should be acknowledged. Firstly, this study was conducted with a sample collected online, meaning that only school psychologists with internet access who felt comfortable with web surveys could participate. The invitation was sent to the school director, who forwarded the link to the questionnaire to the psychologists, but there was no support for the fulfillment of the questionnaire. Secondly, despite the selection of school psychologists as participants, who were intentionally chosen to answer our research questions, we agree that other educational professionals may have different perspectives and describe practices in other ways. Other stakeholders may be considered in subsequent studies. Thirdly, although a general definition of PBIS/Schoolwide PBIS was provided in the questionnaire, based on Sugai and Horner (2009), the findings suggest that school psychologists have limited knowledge of schoolwide PBIS, which might have influenced their responses to questions about PBIS implementation.

Future studies should analyze the characteristics of schools that implement the Schoolwide PBIS framework to learn about the strategies developed, the challenges that appear, and how schools deal with barriers to implementation. Specifically, it is essential to understand school psychologists’ perceptions of constraints and facilitating factors in implementing Schoolwide PBIS. Qualitative methodology is relevant to conducting a comprehensive approach regarding the conditions and variables that facilitate Schoolwide PBIS practices in school contexts. Understanding how educational professionals in different countries see Schoolwide PBIS and how it is implemented is crucial to increasing the cultural relevance and efficacy of the framework.

## Figures and Tables

**Table 1:** Participants’ sociodemographic data.

	*n* (%)	M (DP)	Range
Age		45.30 (8.99)	26–68
Gender			
Female	344 (91.7)		
Male	31 (8.3)		
Qualifications			
Bachelor	163 (43.5)		
Master	202 (53.9)		
PhD	10 (2.7)		
Specialist assigned by the Portuguese Psychology Association			
Clinic and health Psychology	129 (34.4)		
Educational Psychology	229 (61.1)		
Work, social, and organizational Psychology	10 (2.7)		
Missing information	7 (1.8)		
Years of experience at their school			
Up to 5 years	151 (40.3)		
6–10 years	96 (25.6)		
More than 11 years	127 (33.9)		
Missing information	1 (0.3)		

**Table 2:** School-level characteristics.

	*n* (%)
School context	
Urban	148 (39.5)
Rural	75 (20.0)
Semi-urban	152 (40.5)
Level of teaching	
Early childhood	298 (79.5)
1st level	309 (82.4)
2nd level	320 (85.3)
3rd level	350 (93.3)
Secondary	224 (59.7)
Type of school	
Non-clustered	58 (15.5)
Clustered	317 (84.5)
Specific Schools	
Disadvantage school contexts	63 (16.8)
Professional schools	49 (13.1)
Artistic schools	18 (4.8)
Regular schools	245 (65.3)
Number of students	
Less than 500	47 (12.5)
501–1000	73 (19.5)
1001–1500	94 (25.1)
1501–2000	81 (21.6)
More than 2000	80 (21.3)

**Table 3:** Number and percentage (%) of schools with practices related to Schoolwide PBIS (N = 375).

Item		Non-Existent	Emergent	In Progress	Sustained	Do Not Know
Expectations defined						
	Defines and disseminates expectations and examples of expected behaviors for students in different settings of the school	47 (12.5)	72 (19.2)	126 (33.6)	120 (32.0)	10 (2.7)
	Defines and disseminates expectations and examples of expected behaviors for school professionals in different settings of the school	73 (19.5)	77 (20.5)	109 (29.1)	98 (26.1)	18 (4.8)
Behavioral expectations taught						
	The expected social behaviors are taught directly and explicitly in the classroom	4 (1.1)	30 (8.0)	101 (26.9)	224 (59.7)	16 (4.3)
	The expected social behaviors are taught directly and explicitly in the whole school	13 (3.5)	50 (13.3)	140 (37.3)	161 (42.9)	11 (2.9)
System for rewarding behavioral expectations						
	Once taught, positive behaviors are remembered and reinforced	15 (4.0)	69 (18.4)	133 (35.5)	140 (37.3)	18 (4.8)
	Positive behaviors are recognized and valued	84 (22.4)	71 (18.9)	98 (26.1)	97 (25.9)	25 (6.7)
System for responding to behavioral violations						
	The school has clear procedures for responding to behavioral problems with students	9 (2.4)	34 (9.1)	120 (32.0)	207 (55.2)	5 (1.3)
	The school’s procedures for dealing with student’s behavior are implemented consistently	10 (2.7)	40 (10.7)	143 (38.1)	170 (45.3)	12 (3.2)
Shared responsibilities						
	The school involves teachers and staff in developing a vision oriented to the promotion of positive behavior	14 (3.7)	56 (14.9)	201 (53.6)	100 (26.7)	4 (1.1)
	The school involves families in developing a vision oriented to the promotion of positive behavior	16 (4.3)	81 (21.6)	191 (50.9)	85 (22.7)	2 (0.5)
	The students are involved in decision-making related to behavior	20 (5.3)	61 (16.3)	159 (42.4)	126 (33.6)	9 (2.4)
Management						
	The school has a team responsible for designing, implementing, and monitoring positive behavior interventions	60 (16.0)	78 (20.8)	130 (34.7)	93 (24.8)	14 (3.7)
	The school has a written document that guides school professionals’ practices related to the promotion of positive behavior	113 (30.1)	63 (16.8)	86 (22.9)	84 (22.4)	29 (7.7)
Monitoring and evaluation						
	The school collects and analyses periodically behavioral indicators	45 (12.0)	65 (17.3)	124 (33.1)	118 (31.5)	23 (6.1)
	The school promotes the dissemination of monitoring results of behavioral indicators to teachers and staff	69 (18.4)	64 (17.1)	116 (30.9)	98 (26.1)	28 (7.5)
	The school promotes the dissemination of monitoring results of behavioral indicators to students	77 (20.5)	77 (20.5)	110 (29.3)	77 (20.5)	34 (9.1)
	The school promotes the dissemination of monitoring results of behavioral indicators to families	75 (20.0)	77 (20.5)	114 (30.4)	76 (20.3)	33 (8.8)
Data-driven decision-making						
	The school makes organizational decisions considering the analysis of the behavioral indicators	58 (15.5)	70 (18.7)	144 (38.4)	78 (20.8)	25 (6.7)
	The school makes pedagogical decisions considering the analysis of the behavioral indicators	35 (9.3)	69 (18.4)	138 (36.8)	113 (30.1)	20 (5.3)
Partnerships						
	Partnerships are developed to support the design, implementation, and monitoring of positive behavioral interventions	55 (14.7)	78 (20.8)	121 (32.3)	91 (32.3)	30 (8.0)
Support to implementation						
	Teachers receive training and/or ongoing support for the implementation of behavioral interventions and supports	29 (7.7)	104 (27.7)	134 (35.7)	78 (20.8)	30 (8.0)
	Administrative staff receive training and/or ongoing support for the implementation of behavioral interventions and supports	49 (13.1)	113 (30.1)	121 (32.3)	56 (14.9)	36 (9.6)
	Auxiliary staff receive training and/or ongoing support for the implementation of behavioral interventions and supports	34 (9.1)	111 (29.6)	140 (37.3)	61 (16.3)	29 (7.7)
Projects, interventions, and support						
	The school has projects and interventions to promote positive behaviors	13 (3.5)	41 (10.9)	144 (38.4)	168 (44.8)	9 (2.4)
	The school has projects and interventions to manage and resolve behavior problems of specific groups of students	19 (5.1)	55 (14.7)	149 (39.7)	145 (38.7)	7 (1.9)
	The school has specific interventions to respond to behavioral problems	6 (1.6)	47 (12.5)	141 (37.6)	175 (46.7)	6 (1.6)

**Table 4:** Frequencies of self-reported knowledge of school psychologists regarding Schoolwide PBIS framework (N = 365).

		Level of Knowledge		
Knowledge About…	Missing Information	None or Little	Some	Good and Very Good
Schoolwide PBIS framework	1 (3.0)	215 (57.3)	121 (32.3)	28 (7.4)
Main features of the Schoolwide PBIS framework	1 (3.0)	227 (60.5)	109 (29.0)	28 (7.5)

## Data Availability

Data from this study are not shared publicly.
